# Investigation of *Sarcocystis* spp. in slaughtered cattle and sheep by peptic digestion and histological examination in Sulaimani Province, Iraq

**DOI:** 10.14202/vetworld.2021.468-474

**Published:** 2021-02-22

**Authors:** Shadan Hassan Abdullah

**Affiliations:** Department of Microbiology, College of Veterinary Medicine, Sulaimani University, Sulaimaniyah, Iraq

**Keywords:** cattle, muscle samples, prevalence rate, *Sarcocystis*, sheep

## Abstract

**Background and Aim:**

Sarcocystosis is a zoonotic infection caused by various species of *Sarcocystis* organisms with a worldwide geographic distribution. This study investigated the presence of *Sarcocystis* organisms in cattle and sheep slaughtered at an abattoir in Sulaimani Province in North Iraq.

**Materials and Methods:**

A total of 130 muscle samples were collected during May, June, and July of 2020, including 80 samples from sheep and 50 samples from cattle. Samples were examined visually for macrosarcocysts. The peptic digestion method was used to analyze fresh muscle tissue samples for detecting microsarcocysts followed by microscopic examination. Furthermore, muscle samples were fixed and stained with hematoxylin and eosin for histopathological examination.

**Results:**

In the gross examination, macroscopic cysts were not detected in both cattle and sheep; hence, all the prevalence data were obtained through microscopic observation of muscle samples. The peptic digestion method revealed the presence of banana-shaped bradyzoites in 90% and 92.5% of slaughtered cattle and sheep muscle samples, respectively. Organ-wise prevalence revealed that 95% and 92% of esophageal samples of sheep and cattle contained *Sarcocystis* spp., respectively Moreover, 90% and 88% of sheep and cattle diaphragms were respectively infected. Histopathological examination of tissue sections revealed two morphologically distinct types of microsarcocysts, including thin-walled and thick-walled, in both sheep and cattle.

**Conclusion:**

The suspected *Sarcocystis* spp. were *Sarcocystis tenella* and *Sarcocystis arieticanis* in sheep and *Sarcocystis cruzi* and *Sarcocystis bovifelis* or *Sarcocystis hominis* in cattle. Infective stages of different *Sarcocystis* spp. are widespread in the study area environment.

## Introduction

Sarcocystosis is caused by unicellular coccidian protozoan parasites belonging to *Sarcocystis* species [1,2]. This genus consists of more than 189 species with global distribution [3]. The majority of the known *Sarcocystis* spp. appear to be intermediate host-specific [4].

A wide range of vertebrates, including mammals, birds, and fish, are infected by *Sarcocystis* spp. Merogony and cyst formation (asexual stage) occur in the intermediate host, whereas gametogony and sporogony (sexual stages) occur in the definitive host [5].

Definitive hosts are infected through the ingestion of *Sarcocystis* in muscle tissues, which include carnivorous predators, scavengers, as well as humans. Intermediate hosts become infected through the ingestion of oocysts/sporocysts present in feed or water contaminated by fecal materials, which typically consist of herbivores and omnivores [4].

Most of the pathogenic *Sarcocystis* spp. cause disease only in their intermediate hosts. In general, *Sarcocystis* spp. transmitted by canids or primates are more pathogenic than those transmitted by felids [5].

Although the majority of *Sarcocystis* infections are asymptomatic for intermediate hosts, acute cases have also been reported with clinical signs of weakness, anorexia, diarrhea, weight loss, muscle twitching, and, in rare cases, death of the infected animal [6]. These symptoms occur during the development of the second generation of intravascular meronts [7]. The common pathological change associated with sarcocystosis in the intermediate host is the development of tissue cysts containing bradyzoites in the muscle tissues. Occasionally, encephalitis may occur in sheep and horses after infection with *Sarcocystis ovicanis* and *Sarcocystis neurona* [8].

Humans can be infected with intestinal sarcocystosis through the consumption of raw or undercooked beef, pork, and meat products containing the bradyzoites of *Sarcocystis hominis* and *Sarcocystis suihominis* [9]. The transmission of *Sarcocystis cruzi* to humans has also been reported [10]. However, *S. cruzi* has also been reported in sheep. Nevertheless, the possibility of its transmission from sheep to humans requires careful investigation [11].

Infection generally includes transient nonspecific gastrointestinal manifestations. The severity correlates with the number of ingested sarcocysts [1]. The diagnosis of intermediate hosts is commonly possible during necropsy because the disease exhibits either a subclinical or chronic course [12].

It has been reported that sarcocystosis in sheep (*Ovis aries*) is caused by *Sarcocystis tenella*, *Scolopendra gigantea*, *Sarcocystis medusiformis*, and *Sarcocystis arieticanis* [13]. Moreover, *Sarcocystis moulei* and *S. cruzi* have been reported in sheep [11]. At the same time, cattle (*Bos taurus*) are an intermediate host for *Sarcocystis* spp., including *S. cruzi*, *S. hominis*, *Sarcocystis hirsuta*, and *Sarcocystis bovifelis* [14]. Other species include *Sarcocystis sinensis* [15]. Recently, *Sarcocystis heydorni*, *Sarcocystis rommeli*, and *Sarcocystis bovini* have also been identified in bovines [9,14].

*Sarcocystis* spp. can be microscopic, whereas others are macroscopic sarcocysts [13]; macrosarcocysts in striated muscles are large enough to be distinguished by the naked eye, as they occur as elongated cylindrical bodies and milky white-colored cysts embedded in muscular tissues, with length ranging from <5 mm to >10 mm. Two forms of examined macrosarcocysts were present, including fat and thin forms [16]. In sheep, *S. gigantea* and *S. medusiformis* form macroscopic sarcocysts that are transmitted by felids, whereas other *Sarcocystis* spp. transmitted by canines are microscopic [13]. In cattle, mature *S. hirsuta* cysts are macroscopic, whereas cysts of other species are microscopic [4].

Microscopic sarcocysts are generally identified based on the morphology of the cyst wall; *S. cruzi* and *S. heydorni* have thin-walled cysts, and *S. bovini*, *S. bovifelis*, *S. hirsuta*, and *S. hominis* have thick-walled cysts [4,9]. The sarcocysts of *S. tenella* have thick cyst walls, whereas those of *S. arieticanis* have thin cyst walls [17].

The definitive identification of *Sarcocystis* species requires an electron microscope or a molecular detection method [18]. Molecular techniques have been used to differentiate *Sarcocystis* species in cattle and sheep [4,19].

Sarcocystosis, due to its severe economic, medical, and veterinary consequences, is considered as a significant public health issue in several countries [20].

Due to the lack of available data on the prevalence of *Sarcocystis* spp., this study was conducted to investigate the presence of *Sarcocystis* organisms in meat-producing animals (cattle and sheep) slaughtered at an abattoir in Sulaimani Province in the northern region of Iraq.

## Materials and Methods

### Ethical approval

The study is not based on *in vivo* experiments or live animals; the sampling was performed following the slaughtering of animals in the slaughterhouse.

### Study period and location

This study was conducted during May, June, and July of 2020. Muscle samples were collected from the diaphragm and esophagus of slaughtered cattle and sheep of different ages and sexes after inspection of meat at the abattoir in Sulaimani Province. The samples were examined for the presence of *Sarcocystis* spp.

### Sample collection

A total of 130 muscle samples, including 80 from sheep (40 diaphragm and 40 esophagus samples) and 50 from cattle (25 diaphragm and 25 esophagus samples), were collected. Samples were placed in plastic bags, labeled, and transported in a CoolBox to the laboratory for further investigation.

Fresh tissue samples were subjected to gross examination for the presence of macrocysts, and the microsarcocysts were detected using the peptic digestion method, followed by microscopic examination and histological examination of stained muscle samples [21].

### Microscopic examination using peptic digestion method

Muscle samples were minced and subjected to peptic digestion separately using the method described by Dubey *et al*. [12]. Approximately 20 g of each minced sample was processed using 50 mL of the digestion solution, comprising 1.3 g pepsin, 2.5 g NaCl, and 3.5 mL concentrated HCl in a total volume of 500 mL of distilled water.

The suspensions were set at room temperature for 30 min and then filtered using a strainer with gauze. Filtrates were collected in sterile test tubes and centrifuged at 1500 rpm for 15 min, and the sediment was suspended in 0.5 mL of distilled water [22].

The suspension was examined for the presence of *Sarcocystis* bradyzoites under a light microscope at 40×. Free bradyzoites were observed as banana-shaped bodies. In addition, smears were prepared using droplets from the same solution spread on glass slides, fixed with absolute methanol, and stained with 10% Giemsa solution for 5 min. Slides were examined under a microscope at ×100 for detecting *Sarcocystis* bradyzoites [23].

### Histological examination

A total of 16 samples that were positive in the microscopic examination were subjected to histopathological investigation (eight samples from each organ) for both animal species. Slices of muscle samples were preserved, fixed in 10% neutral buffered formalin, and embedded in paraffin blocks for histological examination. The tissues were cut into 3-5 mm thick sections and stained with hematoxylin and eosin (H&E) [24]. Slides were examined for the presence of sarcocysts at 10× and further analyzed at 40× and 100×. For the morphological identification of *Sarcocystis* spp., the cell wall structure was observed [25].

### Statistical analysis

Data analysis was conducted using SPSS Statistic Version 19 (IBM, USA). Chi-square tests were used to determine the association between different organs (inspected esophagus and diaphragms in both cattle and sheep). p<0.05 was considered to be statistically significant.

## Results

The overall prevalence rates of *Sarcocystis* spp. as examined by light microscopy were 90% (45/50) and 92.5% (74/80) in slaughtered cattle and sheep, respectively. There were no significant differences (p>0.05) between the overall *Sarcocystis* detection rates in the examined host species (Table-1).

**Table-1:** Prevalence of microsarcocyst of *Sarcocysts* spp. in slaughtered cattle and sheep at Sulaimani abattoir, north Iraq.

Inspected organs	Examined animal				Total infected

	Cattle		Sheep		
	
	Inspected no.	Infected no. (%)	Inspected no.	Infected no. (%)	
Esophagus	25	23 (92)	40	38 (95)	65
Diaphragm	25	22 (88)	40	36 (90)	65
Total	50	45 (90)	80	74 (92.5)	130

Organ-wise prevalence revealed higher positive rates of *Sarcocystis* spp. in the esophagus than in the diaphragm. Approximately 95% (38/40) of esophageal samples contained *Sarcocystis*, and 90% (36/40) of diaphragm samples were positive in sheep. In cattle also, 92% (23/25) of esophageal samples were positive, and 88% (22/25) of diaphragm samples were positive for *Sarcocystis* spp. However, there were no significant differences (p>0.05) between the examined organs in both cattle and sheep. The distribution of *Sarcocystis* spp. in cattle and sheep muscles is shown in Table-1.

In the gross observation, no macroscopic sarcocysts were found in any of the examined muscle samples representing cattle and sheep. This finding may be due to the variation in risk factors associated with the distribution of *Sarcocystis* infection.

**Figure-1:**
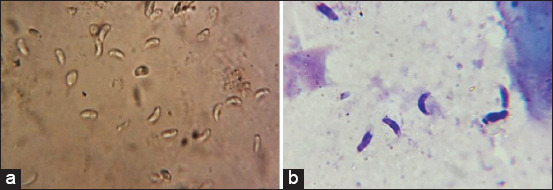
(a) Fresh smear preparation from microcyst, showing crescent-shaped bradyzoites (40×), (b) Giemsa stained from digested muscle samples showing *Sarcocystis* bradyzoites (100×).

In the microscopic observation, the bradyzoites appeared crescent shaped and had pointed anterior end and rounded posterior end as analyzed using the peptic digestion method (Figure-1).

**Figure-2:**
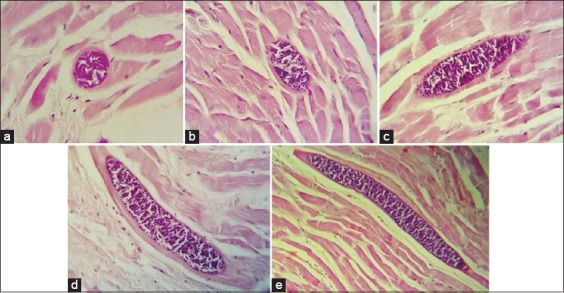
(a-e) Longitudinal section of *Sarcocystis* spp. revealing different shapes of microsarcocyst with hematoxylin and eosin stain of diaphragm muscles (10×).

**Figure-3:**
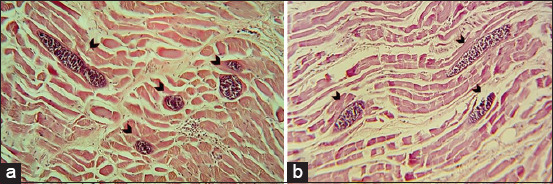
Longitudinal section of unidentified *Sarcocystis* spp. (a and b) Revealing different microsarcocysts with hematoxylin and eosin stain of diaphragm muscles (10×).

Histopathological examination of H&E-stained tissue sections revealed the presence of elongated, spindle-, round-, and ovoid-shaped microscopic tissue cysts packed with banana-shaped bradyzoites (Figure-2). Microscopic observation also revealed variations in the intensity of *Sarcocystis* spp. invasion, varying from single to numerous cysts (Figure-3).

**Figure-4:**
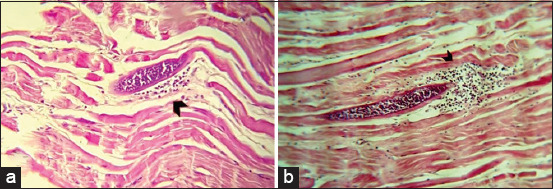
Histological sections from muscle tissue with an unidentified *Sarcocystis* spp. hematoxylin and eosin stain (a and b) showing inflammatory reaction (10×).

Regarding the pathological changes caused by *Sarcocystis* spp., an inflammatory response around several microcysts was detected in the infected muscle fibers accompanied by infiltration of mononuclear cells and degeneration and necrosis of muscle fibers (Figure-4).

**Figure-5:**
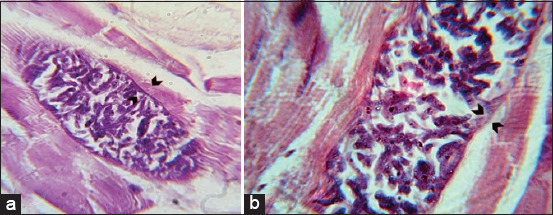
Light microscopic appearance of thin wall microsarcocysts from sheep diaphragm with hematoxylin and eosin stain, (a) 40× and (b) 100×.

**Figure-6:**
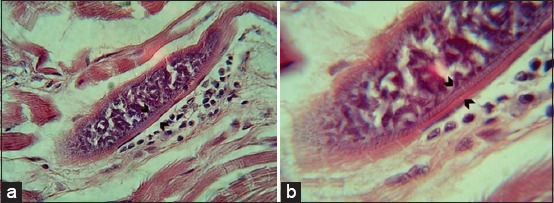
Light microscopic appearance of thick wall microsarcocysts from sheep diaphragm with hematoxylin and eosin stain, (a) 40× and (b) 100×.

**Figure-7:**
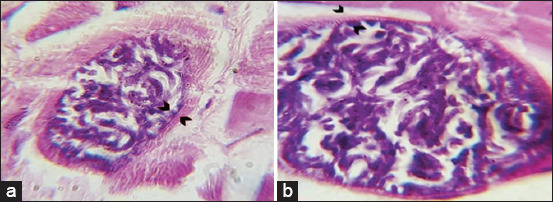
Light microscopic appearance of microsarcocysts from cattle diaphragm with hematoxylin and eosin stain, showing two types of cyst (a) thin wall and (b) thick wall (100×).

**Figure-8:**
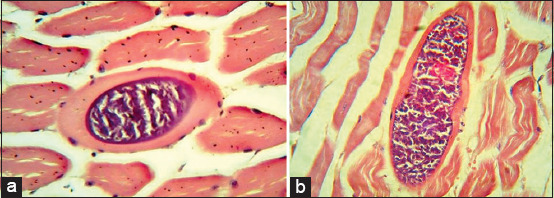
Histological sections of an unidentified *Sarcocystis* spp. showing the microsarcocysts inside the muscle cells from infected cattle (a) esophagus, (b) diaphragm with hematoxylin and eosin stain (40×).

Observation of the microsarcocyst wall under the light microscope revealed two morphologically distinct types, including thin-walled and thick-walled *Sarcocystis* spp., in both sheep and cattle tissue samples (Figures-5-7). Based on the microsarcocyst morphology, the suspected *Sarcocystis* spp. in this study were identified as *S. tenella* and *S. arieticanis* in sheep and *S. cruzi* and *S. bovifelis* or *S. hominis* in cattle. This finding indicated that more significant numbers of *Sarcocystis* spp. were of the thin-walled type in both animals. Sarcocysts were found inside the striated muscle cells including the esophagus, and diaphragm (Figure-8).

## Discussion

*Sarcocystis* is considered as one of the most prevalent livestock parasites, with potential public health importance due to the consumption of undercooked or raw meat. *Sarcocystis* spp. have been isolated from different animals throughout the world. However, to the best of our knowledge, the present study is the first to investigate *Sarcocystis* infection among slaughtered cattle and sheep in Sulaimani Province in North Iraq.

Natural infections of sheep by *Sarcocystis* spp. have been investigated in various countries, with prevalence rates varying from 9.0% to 100%, depending on the detection method [13]. Meanwhile, no macroscopic cysts were detected in the visual examination in the present study, and the overall prevalence was obtained through microscopic examination of muscle tissues, which revealed prevalence rates of 90% and 92.5% of unspecified cysts of *Sarcocystis* spp. by the digestion method in cattle and sheep, respectively.

Diversity in sarcocystosis prevalence has been reported in different geographical areas; for instance, a prevalence rate of 100% was reported by Dehaghi *et al*. [26] and Whaeeb and Faraj [27] in sheep, and prevalence rates of 95.37% and 96.9% were reported by Fukuyo *et al*. [5] and Abuelwafa [28], respectively.

Similarly, the prevalence in bovine muscle was almost 100% in most of the world regions [2]. A prevalence of 100% was reported by Shekarforoush *et al*. [29]. The prevalence rates of 95.5%, 91.33%, and 88.2% were reported by Mavi *et al*. [30], Mounika *et al*. [31], and Shahraki *et al*. [32], respectively.

In the present study, examination of the esophagus and diaphragm muscles revealed higher prevalence rates of 92% and 95% in the esophagus than those of 88% and 90% in the diaphragm for cattle and sheep, respectively. A similar finding was also reported by Dehaghi *et al*. [26] and Ahmed *et al*. [33]. Positive rates of 91% and 58% in cattle esophagus and diaphragm were also reported by Nourani *et al*. [34].

In contrast to our findings, high prevalence rates of *Sarcocystis* spp. of 100% and 93.2% in the diaphragm were reported by Fukuyo *et al*. [5] and Bahari *et al*. [35], respectively, and also by Dubey and Livingston [20] who reported the prevalence rates of 22.4% in the diaphragm and 0.23% in the esophagus of sheep.

*Sarcocystis* was also detected in cattle heart at prevalence rates of 100% and 17.9% from Brazil and Romania by Ferreira *et al*. [36] and Imre *et al*. [37], respectively.

Variations in the prevalence rates of *Sarcocystis* spp. depend on several factors such as management conditions, presence of dogs and cats in the surrounding area, and the number of sporocysts disseminated by them. Survivability of sarcocysts in the environment is another factor affecting the prevalence of *Sarcocystis* controlled by climatic conditions such as temperature, rainfall, and humidity [38].

The present study results showed that the pepsin digestion technique is a sensitive method for investigating the presence of *Sarcocystis* in slaughtered animals [32,39], which produced the highest positive rate. This technique is also simpler and faster than other conventional procedures [16]. However, *Sarcocystis* spp. cannot be appropriately identified using this method.

Similar to the present study result, no macroscopic sarcocysts were observed by Shekarforoush *et al*. [29], Nourani *et al*. [34], and Shahraki *et al*. [32], although low prevalence rates of 17.08% and 7.5% were reported by Farhang-Pajuh *et al*. [16] and Ahmed *et al*. [33] in sheep and cattle, respectively.

Macrosarcocyst species are considered as symptomatic. However, they can affect the quality of meat and lead to economic losses [19] in the form of subsequent downgrading, partial rejection, or even condemnation of the animal carcasses at slaughterhouses [28].

Primarily, felids comprise the definitive hosts for macrosarcocyst forming species. Due to lesser contact with felids, lesser numbers of oocytes are shed, which thus explain the low prevalence of macroscopic cysts [30].

Dogs play a more significant role than cats in the transmission of sarcocystosis in farm animals, which might be attributed to the close association with dogs that are retained for herd protection. Moreover, dogs can easily access the offal of infected sheep, thus making it easier for them to transmit *Sarcocystis* [28].

In the present study, histopathological examination of H&E-stained tissue sections revealed the presence of two morphologically distinct microscopic types of sarcocysts. Consistent with El-Morsey *et al*. [17], the observed microsarcocysts in sheep muscle tissues were of thin-walled and thick-walled types, which belong to *S. arieticanis* and *S. tenella*, respectively, and those of *S. tenella* had striated thick-walled sarcocysts (Figure-7). Moreover, the bovine species *S. cruzi* has been reported to have thin-walled microsarcocysts detected in sheep [11]. Similarly, both *S. tenella* and *S. arieticanis* were reported through light microscopic examination by Hu *et al*. [40].

*Sarcocystis* spp. in cattle were also of two types. *S. cruzi* and *S. heydorni* have thin-walled cysts, and *S. bovini*, *S. bovifelis*, and *S. hominis* have thick-walled cysts [25].

In this regard, based on the morphological appearance, the thin-walled microsarcocysts observed in the present study might be *S. cruzi* and *S. heydorni*. Moreover, the thick-walled type might belong to *S. hominis*, *S. bovini*, *S. sinensis*, and *S. bovifelis*, as these species are very similar morphologically, and their cyst walls are provided with finger-like protrusions [41]. The cysts cannot be distinguished by light microscopy, whereas a few differences can be identified by transmission electron microscopy [4,6].

Based on the histological examination of the microsarcocyst wall, the suspected *Sarcocystis* spp. were identified as *S. tenella* and *S. arieticanis* in sheep and *S. cruzi* and *S. bovifelis* or *S. hominis* in cattle, as the availability of other species was inadequate.

Based on the data reported by Nourani *et al*. [34], the thin-walled sarcocysts in cattle were consistent with *S. cruzi* with higher prevalence, and the thick-walled sarcocysts could represent *S. hominis* with lower prevalence. Both *S. cruzi* and *S. tenella* were found at higher prevalence rates of 100% and 80% by Daryani *et al*. [20] and Rahdar and Kardooni [42], respectively.

Both intermediate and final hosts can harbor one or more *Sarcocystis* spp. [28], which is consistent with the present study finding in which both thin- and thick-walled microsarcocysts were observed in the same muscle tissue section.

*Sarcocystis* spp. cyst invasions in the muscle tissue can produce inflammation around the muscle fibers and between the cysts, showing mononuclear cells infiltration (neutrophils, lymphocytes, eosinophils, and plasma cells) and degeneration and necrosis of muscle fibers [25]. Consistently, inflammatory reactions were also detected around some of the observed microsarcocysts in the present study (Figure-4).

Identification of *Sarcocystis* spp. is not possible using a microscopic analysis [25]. Therefore, the exact identification and differentiation of *Sarcocystis* spp. in slaughtered animals necessitate other investigations using accurate detection methodologies, including molecular procedures.

Identification of *Sarcocystis* spp. through molecular characterization is a fundamental approach to evaluate the pathogenic species involved in cattle and sheep infections that consequently cause economic losses. In addition, identifying the final host responsible for shedding each of *Sarcocystis* spp. and evaluating the risk of zoonotic infections are essential steps in managing the disease and designing control programs.

## Conclusion

This study reports the presence of *Sarcocystis* spp. in both sheep and cattle from Sulaimani Province. The prevalence was primarily related to the presence of microsarcocysts. Therefore, meat should be cooked sufficiently or frozen before use to prevent health hazards to the consumer. The pepsin digestion procedure was found to be a sensitive method for investigating unidentified *Sarcocystis* spp. Based on the morphology of the microsarcocyst wall, the suspected *Sarcocystis* spp. recovered in this study were identified as *S. tenella* and *S. arieticanis* in sheep and *S. cruzi* and *S. bovifelis* or *S. hominis* in cattle.

## Author’s Contributions

SHA designed the study and contributed to the collection of muscle samples, the practical parts, analysis of data, and writing and organization of the paper. SHA has read and approved the final manuscript.
